# Identification of important genes related to anoikis in acute myocardial infarction

**DOI:** 10.1111/jcmm.18264

**Published:** 2024-03-25

**Authors:** Puwei Song, Yasen Yakufujiang, Jianghui Zhou, Shaorui Gu, Wenli Wang, Zhengyuan Huo

**Affiliations:** ^1^ Department of Thoracic‐Cardiovascular Surgery, Shanghai Tongji Hospital, School of Medicine Tongji University Shanghai China

**Keywords:** AMI, anoikis, Heart, myocardial infarction

## Abstract

Acute myocardial infarction (AMI) increasingly precipitates severe heart failure, with diagnoses now extending to progressively younger demographics. The focus of this study was to pinpoint critical genes linked to both AMI and anoikis, thereby unveiling potential novel biomarkers for AMI detection and intervention. Differential analysis was performed to identify significant differences in expression, and gene functionality was explored. Weighted gene coexpression network analysis (WGCNA) was used to construct gene coexpression networks. Immunoinfiltration analysis quantified immune cell abundance. Protein–protein interaction (PPI) analysis identified the proteins that interact with theanoikis. MCODE identified key functional modules. Drug enrichment analysis identified relevant compounds explored in the DsigDB. Through WGCNA, 13 key genes associated with anoikis and differentially expressed genes were identified. GO and KEGG pathway enrichment revealed the regulation of apoptotic signalling pathways and negative regulation of anoikis. PPI network analysis was also conducted, and 10 hub genes, such as IL1B, ZAP70, LCK, FASLG, CD4, LRP1, CDH2, MERTK, APOE and VTN were identified. IL1B were correlated with macrophages, mast cells, neutrophils and Tcells in MI, and the most common predicted medications were roxithromycin, NSC267099 and alsterpaullone. This study identified key genes associated with AMI and anoikis, highlighting their role in immune infiltration, diagnosis and medication prediction. These findings provide valuable insights into potential biomarkers and therapeutic targets for AMI.

## 1 INTRODUCTION

Acute myocardial infarction (AMI) is a clinical syndrome that arises from abnormalities in the coronary arteries, specifically the rupture of atherosclerotic plaques within the arterial wall. In cases of acute artery occlusion, there is complete blockage of blood flow, impeding normal circulation within the heart, which leads to severe ischemia and hypoxia in the affected myocardium resulting in cardiac damage and subsequent mortality.^1^ In the acute phase, adverse ventricular remodelling occurs, leading to reduced cardiac function, heart failure and increased mortality.^2^ Additionally, there is myocardial hypertrophy and deposition of interstitial fibrosis in the noninfarcted region, which further contributes to left ventricular remodelling and heart failure.^3^

Anoikis is a term that describes the process of apoptosis that is triggered by the detachment of cells from the extracellular matrix.^4^ It not only plays a crucial role in regulating cell growth, survival and other vital biological functions but also contributes significantly to the pathogenesis of certain cardiovascular disorders.^5^ In patients with AMI, the integrity of the extracellular matrix within the plaque influences thrombus formation, thereby promoting the development of coronary atherosclerosis‐induced AMI.^6^ Recent research findings indicate that the occurrence of anoikis might play a significant role in the development and progression of diverse cardiovascular diseases, including AMI. Elucidating the intricate mechanisms underlying anoikis can provide a foundation for the design and implementation of novel therapeutic approaches. This knowledge empowers researchers and healthcare professionals to explore innovative strategies to prevent or mitigate the impact of cardiovascular diseases, particularly AMI.

To better understand the underlying mechanisms of AMI at the microscopic level and identify crucial biomarkers for diagnosis and therapy evaluation, it is essential to identify distinct gene expression patterns associated with this disease.^7^ By revealing the specific gene expression patterns implicated in AMI, we can gain valuable insights into pathological processes and subsequently develop effective diagnostic and therapeutic strategies. Several bioinformatics software and databases have been developed to facilitate the identification of disease‐associated pathways. Prominent examples of these tools include weighted gene coexpression network analysis (WGCNA), Kyoto Encyclopedia of Genes and Genomes (KEGG) enrichment analysis, and gene set enrichment analysis. These computational resources provide researchers with valuable means to explore and elucidate the molecular pathways that are involved in disease processes.

In this study, a comprehensive analysis was conducted to identify differentially expressed genes (DEGs) by examining data from two distinct groups. WGCNA can be effectively utilized to pinpoint the most relevant modules for AMI, thereby significantly reducing the number of genes requiring screening. Thus, several hub genes related to anoikis that hold promising potential as diagnostic markers for AMI and immune infiltration and for drug prediction were identified. These findings will contribute to our understanding of the underlying mechanisms associated with the risk of AMI.

## 2 METHOD

### 2.1 Data source

The expression data of all the measured genes in the dataset GSE60993, GSE24519 and GSE109048 were downloaded from the Gene Expression Omnibus (GEO) database. GSE60993, GSE24519 were the text set and GSE109048 were utilized to validate the result. For the merger of multiple datasets, we first used the R package in silicoMerging^8^ to combine them. Then, we applied the methods of Johnson WE et al (adjusting batch effects in microarray expression data using empirical Bayes methods)^9^ to remove batch effects.

### 2.2 Differentially expressed gene identification

First, the data of the merged dataset were read using R software (version 3.6.1) and preprocessed it for batch correction and normalization. We subsequently performed DEG analysis screening using the ‘limma’ package. After significance analysis of the expression levels, the ‘pheatmap’ and ‘ggplot2’ R packages were used to construct volcano plots and DEG expression heatmaps, respectively.^10^, ^11^

### 2.3 Weighted gene coexpression network analysis

WGCNA, a systematic approach to biology, is frequently used to characterize genetic association patterns between diverse samples. It is used to find highly synergistic genomes. It can be used to find candidate markers based on the interconnectedness of genomes and the association of genomes with phenotypes.^12^ Using the ‘WGCNA’ R package, we created a gene coexpression network for AMI. Finally, we evaluated the relationship between several modules and the pathogenic mechanism of AMI and selected the most relevant module as the key gene according to WGCNA.

### 2.4 Screening of candidate pivotal genes and functional analysis

These intersecting genes are regarded as candidate hub genes relevant for AMI pathogenesis. KEGG serves as a database resource for systematically analysing gene function.^13^ Subsequently, we performed Gene Ontology (GO) and KEGG enrichment analyses using the ‘clusterProfiler’ R package to determine the potential mechanisms of progression and pathogenesis.^14^, ^15^ We used the gene set enrichment network tool Enrichr based on the Drug Signature Database (DSigDB) to identify potential drugs that significantly interact with genes through PPI network pairs of the screened 10 hub genes. DSigDB is a free web‐based resource repository containing relevant information on drugs and their target genes for GSEA.^16^ DSigDB currently contains a total of 22,527 gene sets, including 17,389 drugs and 19,531 genes.^17^, ^18^ An adjusted *p* value of <0.05 was set as the statistical criteria for identifying drugs significantly associated with target genes.

### 2.5 Protein–protein interaction (PPI) network of the hub genes

The molecular interaction and PPI networks were predicted and displayed using the Search Tool for the Retrieval of Interacting Genes/Proteins (STRING) and Cytoscape software platforms. The degree method used in Cytoscape software was used to determine the significance of genes within PPI networks.^19^, ^20^ We set confidence score >0.4 as the cut‐off criterion. Then, we used Cytoscape software (version 3.6.1) to conduct the PPI network visualization. The APP Molecular Complex Detection (MCODE) (version 1.5.1) was used to identify the most significant module in the networks. The selection criteria were chosen as follows: MCODE scores >5, node score cut‐off = 0.2, degree cut‐off = 2, Max depth = 100 and k‐score = 2.

### 2.6 Immune cell analysis

To investigate the function of immune cells in AMI, we evaluated the level of immune cell infiltration of 22 immunocytes in the AMI group by CIBERSORT analysis.^21^ We also assessed the level of immune cell infiltration of 22 immunocytes in the AMI group using CIBERSORT analysis to study the role of immune cells in AMI.^21^

## 3 RESULTS

### 3.1 The flowchart of bioinformatics analysis of acute myocardial infarction

**FIGURE 1:**
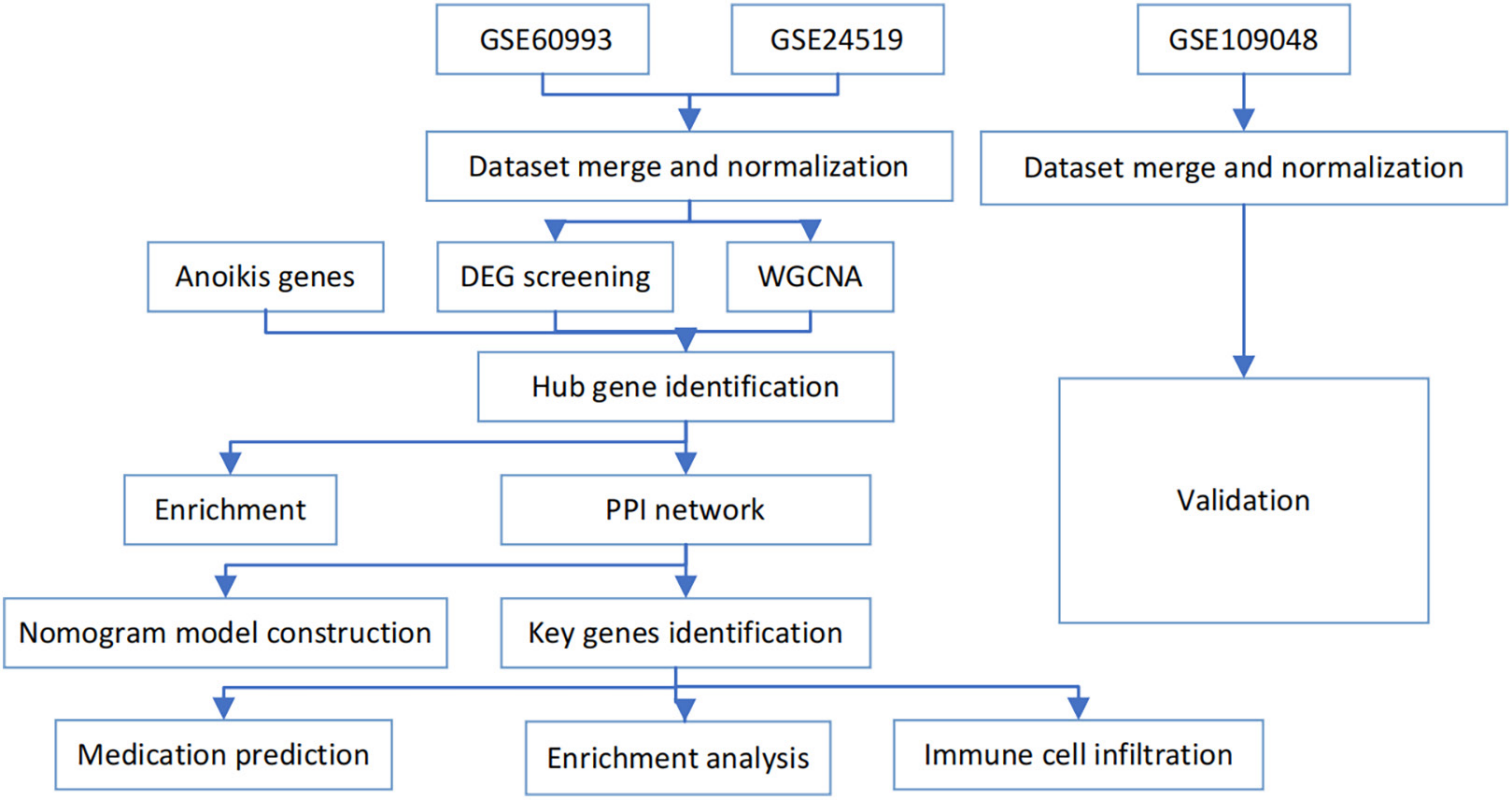
The flowchart of bioinformatics analysis of myocardial infarction. The flowchart shows the main steps and tools used to perform a bioinformatics analysis of RNA‐seq data from human samples with or without acute myocardial infarction, which is a condition caused by the blockage of blood flow to the heart muscle.

Figure 1 shows the main steps and tools used to perform a bioinformatics analysis of RNA‐seq data from human samples with or without AMI, which is a condition caused by the blockage of blood flow to the heart muscle.

### 3.2 Dataset normalization and DEG screening

The acute myocardial infarction datasets (GSE60993 and GSE24519) were initially acquired from the GEO database. The gene array datasets were subsequently normalized and merged to create a comprehensive superarray dataset. This merged dataset included 60 samples of peripheral blood obtained from patients diagnosed with AMI and 11 samples of peripheral blood obtained from normal patients.

**FIGURE 2:**
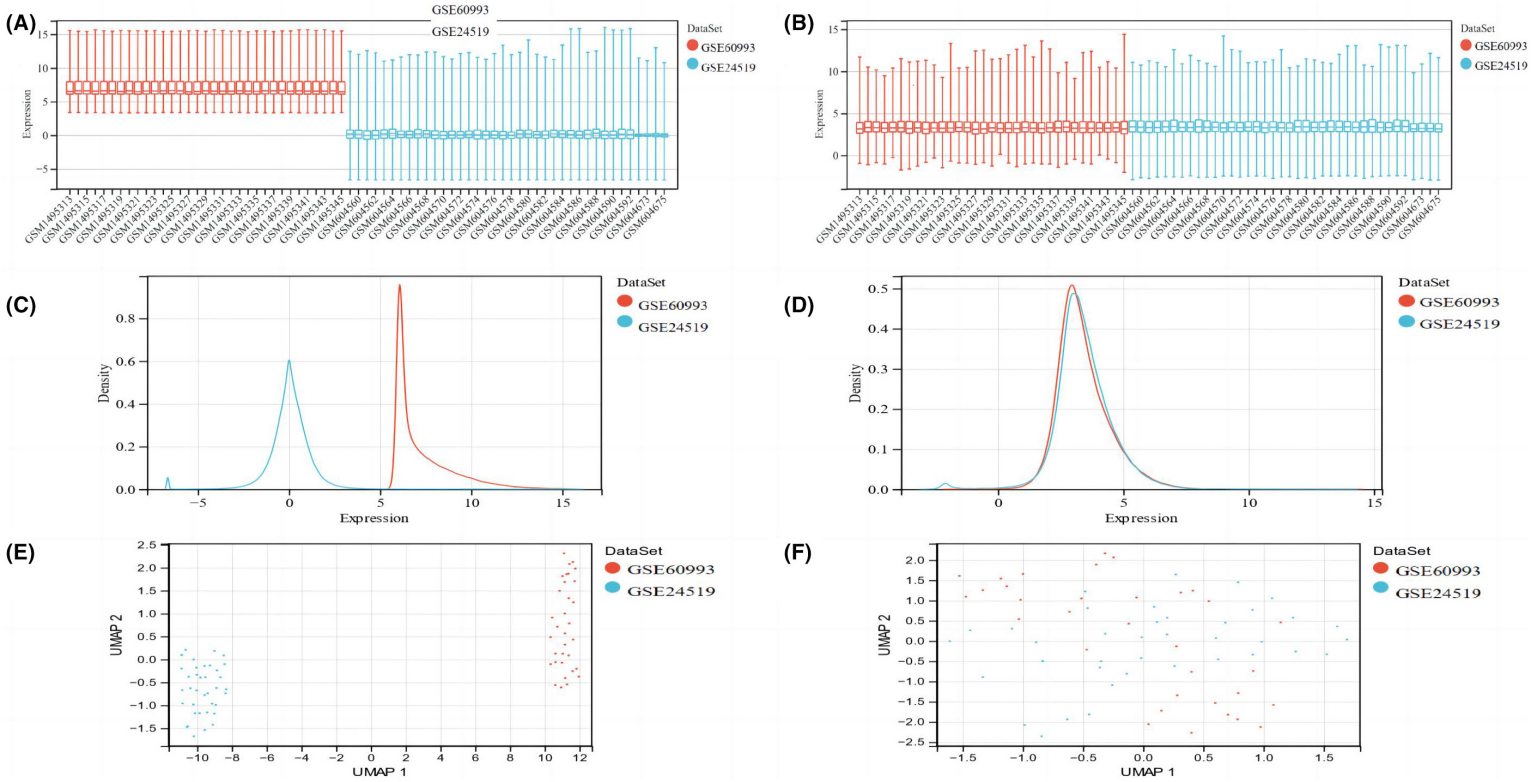
Genes differentially expressed between the acute myocardial infarct and normal groups. (A–D) According to the box plots and density plots, the sample distributions of the datasets before the removal of the batch effect exhibited large differences (Figure 2A and Figure 2C), and after the removal of this effect, the distributions of the datasets tended to be consistent with each other, and the medians were on the same line (Figure 2B and Figure 2D). (E, F) According to the UMAP plot, the samples in each dataset were clustered together before the removal of the batch effect (Figure 2E), and after the removal of the batch effect (Figure 2F), the samples in each dataset were clustered and intertwined with each other.

Based on the analysis of the box plots and density plots, it is evident that the sample distribution of each dataset exhibited substantial dissimilarity prior to elimination of the batch effect (Figure 2A and Figure 2C). This discrepancy strongly suggested the occurrence of a batch effect. However, following the removal of the batch effect, the data distribution between each dataset appeared to converge, with the medians aligning along a common line (Figure 2B and Figure 2D). The UMAP plot clearly showed that the samples within each dataset exhibited clustering prior to elimination of the batch effect (Figure 2E). This observation suggested the occurrence of a batch effect. Conversely, following the removal of the batch effect, the samples from each dataset displayed a clustered and intertwined pattern (Figure 2F), indicating more effective elimination of the batch effect.

### 3.3 Construction of the WGCNA network and identification of AMI‐related modules

**FIGURE 3:**
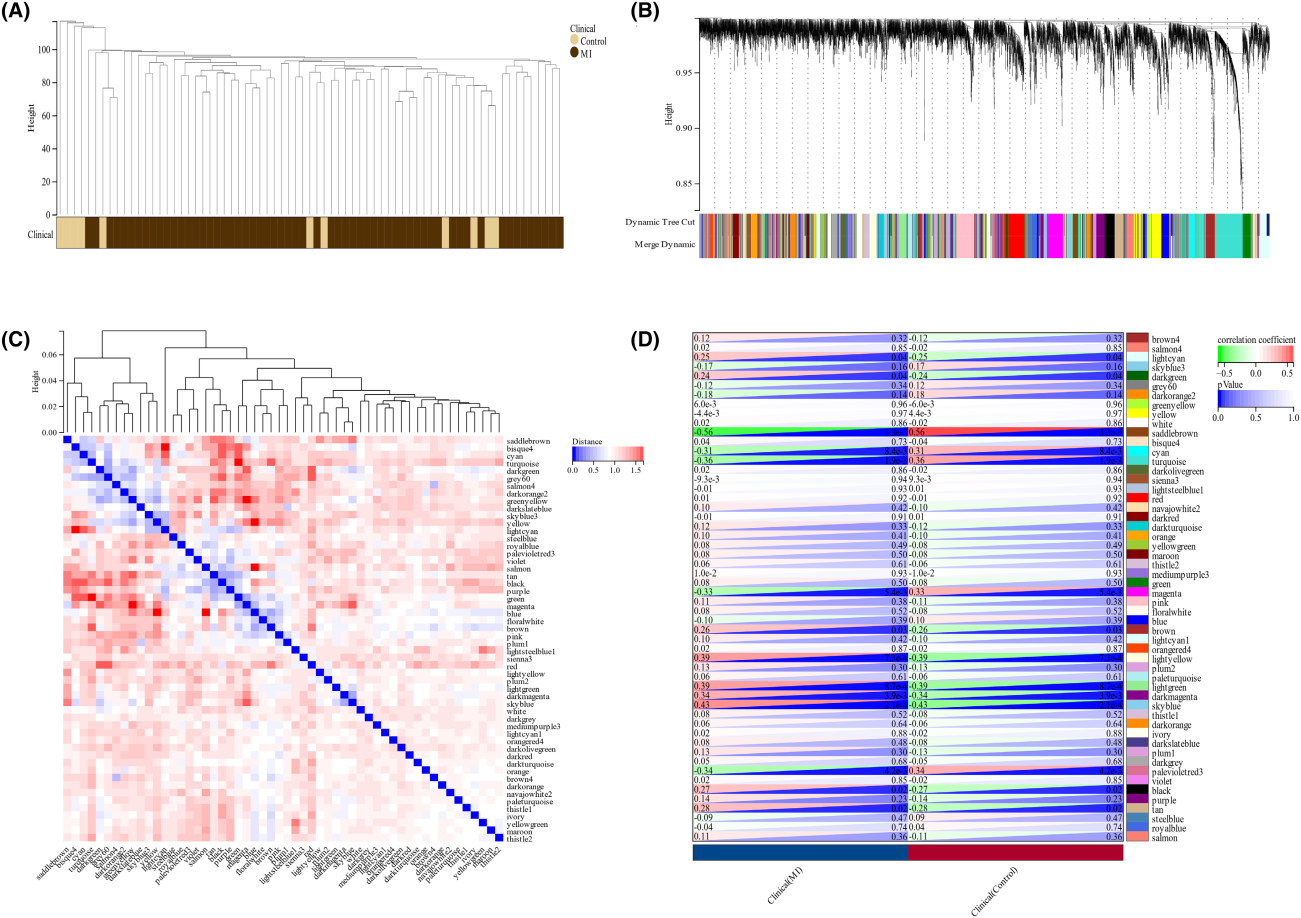
Identification of acute myocardial infarct‐associated gene modules in the GEO dataset using WGCNA. (A, B) Dendrogram of all genes in the merged dataset clustered on the basis of a topological overlap matrix (1‐TOM). Each branch in the clustering tree represents a gene, while coexpression modules are constructed in different colours. (C) the modularity eigenvector clustering of the merged dataset.(D) Module‐trait heatmap of the correlation between the clustering gene module and acute myocardial infarct in the merged dataset. The corresponding correlation coefficient and p value were calculated for each module.

To determine whether the putative gene modules were associated with AMI, we performed WGCNA on all the candidate genes from the datasets related to AMI (Figure 3A,B). In the meanwhile, we applied the modularity eigenvector clustering method to the merged dataset, which facilitated the identification of closely related gene clusters, as depicted in Figure 3C. Module‐trait heatmap of the correlation between the clustering gene module and acute myocardial infarct in the merged dataset (Figure 3D). GO and KEGG analyses and PPI network construction.

**FIGURE 4:**
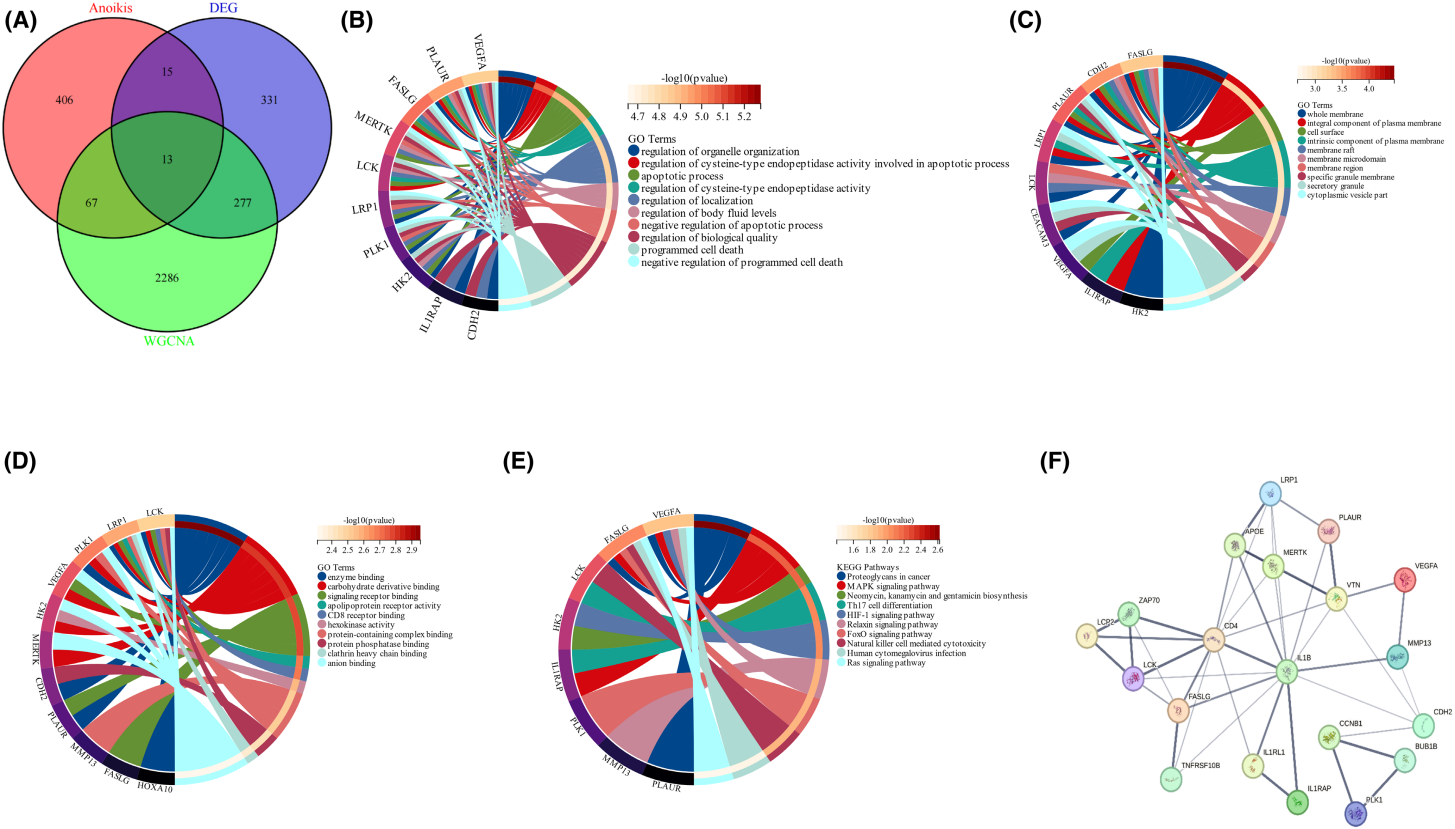
Candidate overlapping genes were screened and enrichment analysed and PPI network was constructed (A) Venn diagram revealing overlapping candidate genes related to anoikis, DEG and genes in the modules correspond with AMI. (B–E) GO and KEGG enrichment analysis of candidate hub genes. Different colours on the outer right circle represent different biological processes and different colours on the outer left circle represent different DEGs. (B). BP, biological process, (C) CC, cellular component, (D) MF, molecular function, (E) KEGG pathway enrichment. (F) PPI network of the overlapping genes.

To identify coexpressed genes, we conducted a thorough analysis within these modules to identify key genes involved in the anoikis process. This analysis was carried out by matching the genes in the modules with a known list of anoikis‐related genes in Gene Card Database and cross‐referencing them with the list of differentially expressed genes (DEGs). A total of 13 overlapping genes were screened as potential genes that may have a significant impact on the development and progression of AMI, as depicted in Figure 4A.

To further investigate the underlying functions of these overlapping genes, GO and KEGG analyses were also conducted. GO enrichment analysis demonstrated that the overlapping genes primarily influenced the biological functions of the regulation of the apoptotic signalling pathway, apolipoprotein receptor activity and enzyme binding (Figure 4B,C,D). KEGG enrichment analysis revealed that the overlapping genes primarily influence the MAPK signalling pathway, Th17 cell differentiation and HIF1signaling pathway (Figure 4E). Then, we constructed a PPI network of the overlapping genes using the STRING tool (Figure 4F).

### 3.4 Hub genes identified and enrichment analysis and medication prediction of hub genes

**FIGURE 5:**
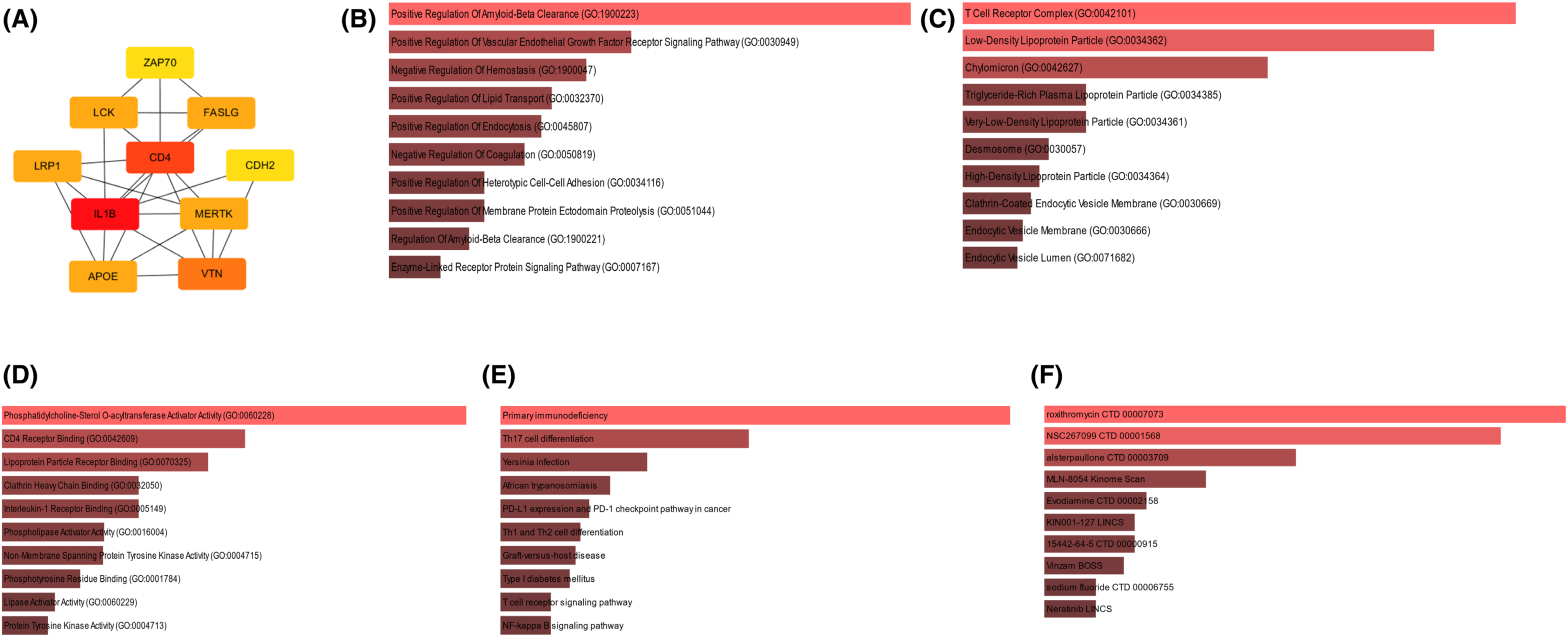
Hub genes identified and enrichment analysed and medication prediction of the hub genes. (A) Top 10 hub genes identified. (B–D) The enriched items in the Gene Ontology analysis of the hub genes. B. BP: biological process, (C) CC, cellular component, (D) MF molecular function, (E) enriched items in the Kyoto Encyclopedia of Genes and Genomes analysis, (F) The top 10 DsigDB prediction enrichment results.

The Cytoscape program was subsequently used to visualize the top 10 hub genes (Figure 5A). In this study, a selection of genes, including IL1B, ZAP70, LCK, FASLG, CD4, LRP1, CDH2, MERTK, APOE and VTN, were identified and categorized. There is a positive correlation between the intensity of the colour and the magnitude of the score. The enrichment analysis of the hub genes was similar to the enrichment analysis of the overlapping genes of the DEGs, turquoise module and modules related to AMI. (Figure 5B,E). The three predicted medications involved were roxithromycin, NSC267099 and alsterpaullone (Figure 5F).

### 3.5 Correlation of immune cell infiltration in AMI patients and hub genes

**FIGURE 6:**
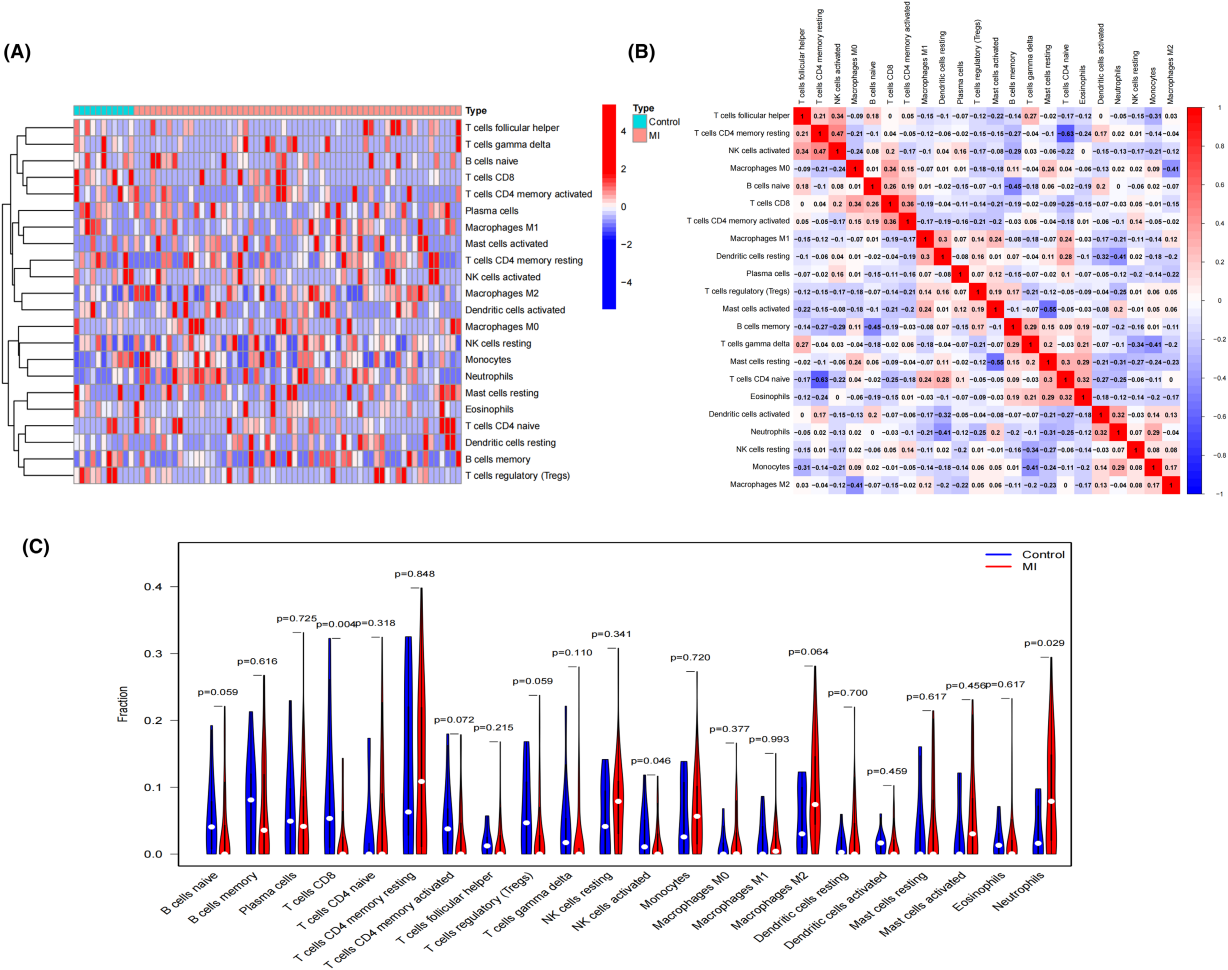
Immune infiltration in acute myocardial infarction. (A) Relative distribution heatmap of immune cells in all acute myocardial infarct samples. (B) Correlation heatmap of immune cells in all acute myocardial infarct samples. (C) Differences in immune cell infiltration between the acute myocardial infarct and control groups.

**FIGURE 7:**
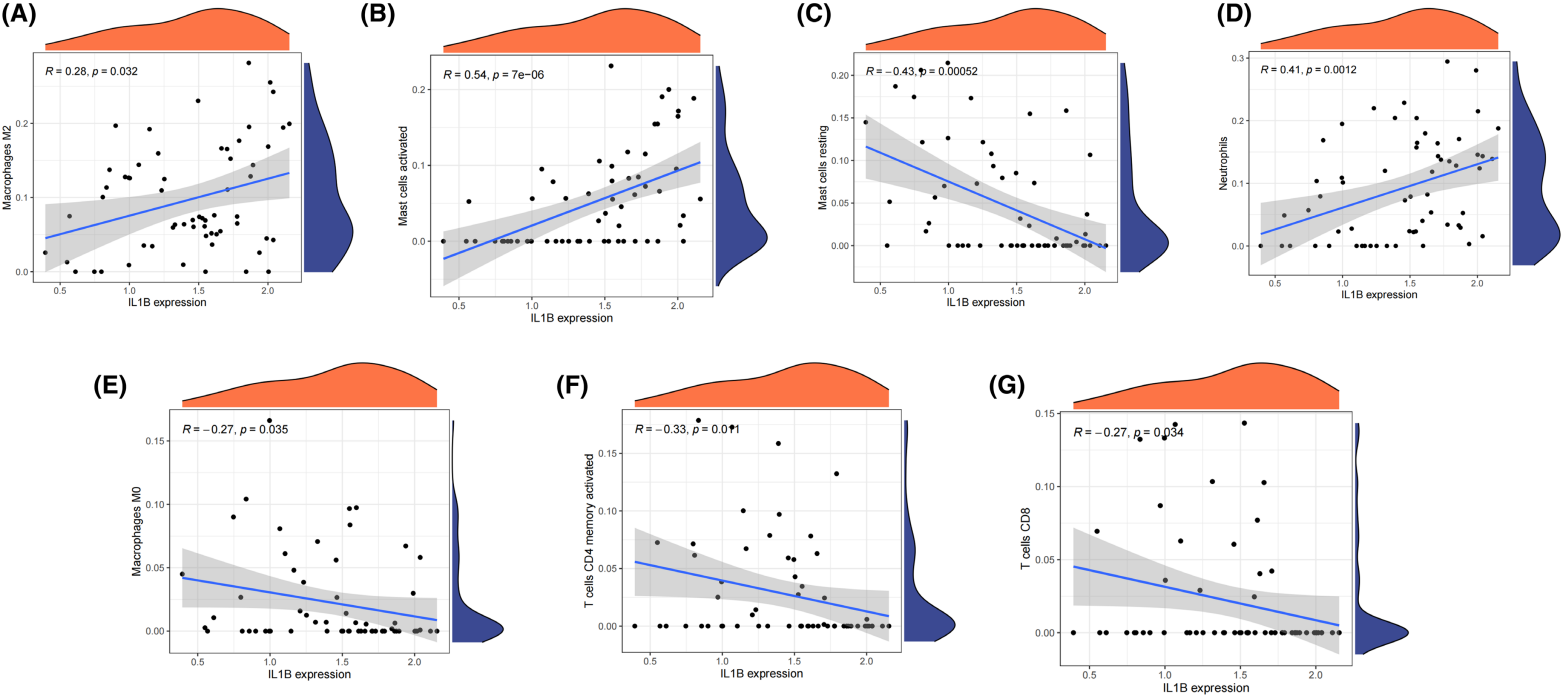
Immuno‐correlation of hub genes IL1B in acute myocardial infarction. (A) Relative correlation between IL1B and Macrophages M2 in the dataset. (B) Relative correlation between IL1B and Mast cells activated in the dataset. (C) Relative correlation of IL1B expression and Mast cell resting in the dataset. (D) Relative correlation of IL1B and Neutrophils in the dataset. (E) Relative correlation between IL1B expression and Macrophages M0 in the dataset. (F) Relative correlation between IL1B and T cell CD4 memory activated in the dataset. (G) Relative correlation between IL1B expression and T cell CD8 in the dataset.

Figure 6A,B depict the relative distribution and correlation heatmap of immune cells in all the AMI patients. These findings indicate that there was greater infiltration of neutrophils in the acute myocardial infarct group than in the control group. Conversely, the infiltration levels of CD8 T cells were lower in the acute myocardial infarct group than in the control group, as shown in Figure 6C. There is a positive association between IL1B and several immunes (Macrophages M2, Mast cells activated and neutrophils) (Figure 7A,B,D). There is a negative association between IL1B and other several immunes (Mast cells resting, Macrophages M0, T cells CD4 memory activated and CD8 T cells) (Figure 7C,E,F,G).

### 3.6 Bioinformatics analysis results validated by GSE109048

**FIGURE 8:**
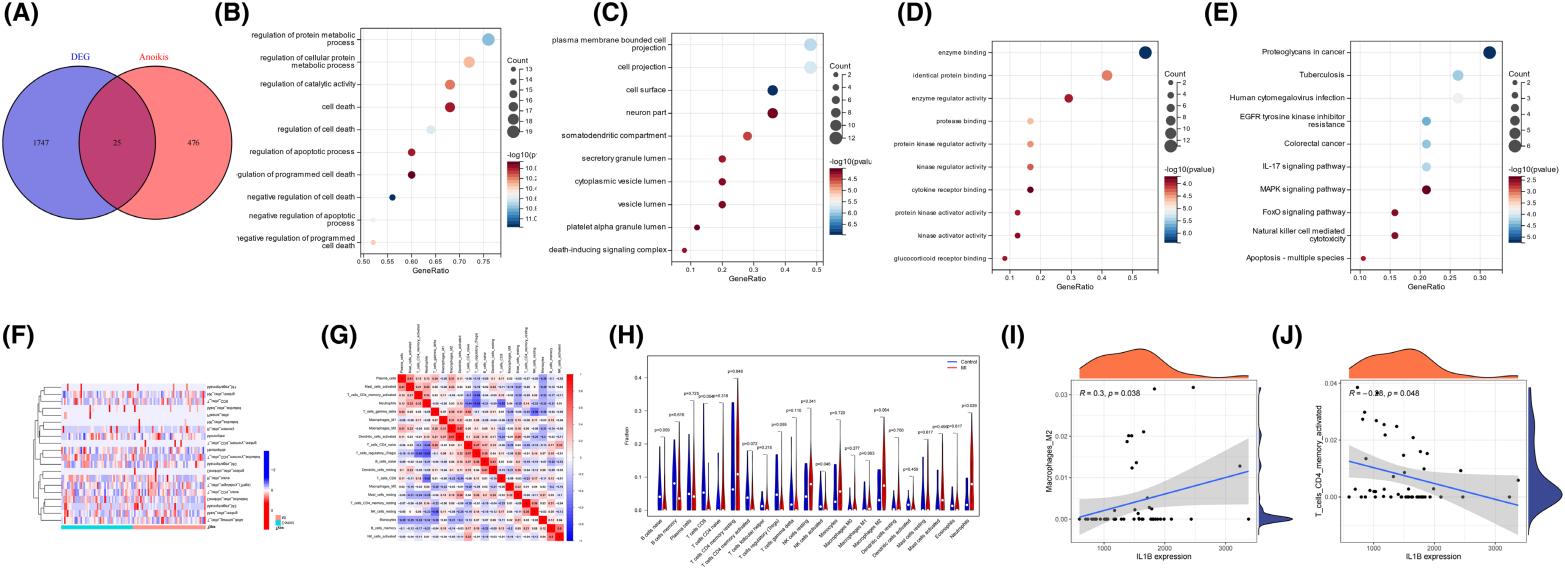
Bioinformatics analysis results validated by GSE109048. (A) Venn diagram revealing overlapping candidate genes related to anoikis and DEG in GSE109048. (B–D) GO enrichment analysis of candidate hub genes. (B). BP, biological process, (C) CC, cellular component, (D) MF, molecular function; (E) KEGG pathway analysis of candidate hub genes. (F) Relative distribution heatmap of immune cells in all acute myocardial infarct samples. (G) Correlation heatmap of immune cells in all acute myocardial infarct samples. (H) Differences in immune cell infiltration between the acute myocardial infarct and control groups. (I) Relative correlation between IL1B and Macrophages M2 in the dataset. (J) Relative correlation between IL1B and T cell CD4 memory activated in the dataset.

Venn diagram revealing overlapping candidate genes related to anoikis and DEG in GSE109048 (Figure 8A). GO and KEGG enrichment analysis of overlapping genes (Figure 8B–8E). Relative distribution heatmap, correlation heatmap of immune cells and differences in immune cell infiltration between the acute myocardial infarct and control groups were coincident with previous result (Figure 8F–H). Relative correlation between IL1B and Macrophages M2, T cell CD4 were coincident with previous result (Figure 8I,J).

## 4 DISCUSSION

AMI is the result of myocardial necrosis caused by acute and prolonged ischemia and hypoxia of the coronary artery and can be life‐threatening. Over the years, there has been steady increasing in the incidence and mortality of AMI, particularly among younger individuals.

Anoikis, the process of apoptosis induced when cells lose their adhesion to the extracellular matrix, plays a critical role in the development of ACS. Previous studies have indicated that neutrophil elastase can degrade basement membrane components, exacerbate endothelial cell injury, and promote anoikis and apoptosis and the levels of this enzyme increase in patients with AMI.^22^

In this study, a bioinformatics analysis was conducted on a comprehensive database merged from GSE60993 and GSE24519. In the WGCNA, a total of 15 modules were identified. Among them, several modules showed strong correlation with MI and 13 key genes within this module that were associated with anoikis‐related DEGs were selected. These genes were subjected to PPI analysis, resulting in the identification of 10 hub genes. The hub genes identified were IL1B, ZAP70, LCK, FASLG, CD4, LRP1, CDH2, MERTK, APOE and VTN and IL1B was the crucial genes among them.IL‐1B is a key pro‐inflammatory cytokine that is associated with the development of atherosclerosis and MI.^23^ IL1B gene polymorphisms affect the risk of MI and ischemic stroke in young adults by modulating the expression of NF‐κB, iNOS, MMP‐2, and Bax.^24^ Biochemical analysis of AMI revealed that IL1B is a biomarker of acute MI and mediates the inflammatory response after acute MI.^25^ Interestingly, in acute MI and ischemic stroke, the expression of APAF1 and IRAK3 was negatively correlated with the abundance of NK cells, while the expression of ATM, CAPN1, IL1B, IL1R1, PRKACA, PRKACB and TNFRSF1A was positively correlated with the abundance of NK cells in MI.^26^ The 511 T single nucleotide polymorphism (SNP) in IL1B was associated with lower release of IL‐1B in human monocytes and lower risk of MI in young adults.^27^

Meanwhile, Hongwei Sun reported overexpression of CDH2 in MI vascular smooth muscle cells compared with that in controls.^28^ Interestingly, the mesenchymal marker CDH2 was downregulated, while the epithelial marker CDH1 was increased during MI, suggesting that EMT‐related signalling rather than the EMT process is associated with AMI.^29^

As to drug enriched, roxithromycin is a macrolide antibiotic that has anti‐inflammatory and immunomodulatory properties. The cardioprotective and endothelial protective effects of roxithromycin have been studied in experimental animals with models of coronary occlusive MI and endothelial dysfunction.^30^ Trials of roxithromycin or azithromycin have demonstrated the role of secondary prevention of ischemic heart disease.^31^ Roxithromycin can be used as a therapeutic intervention for patients with coronary artery disease.^32^ Roxithromycin treatment resulted in a statistically significant reduction of recurrent cardiovascular events.^33^ However, during a 12‐month follow‐up period, roxithromycin treatment of patients with AMI did not reduce the incidence of events.^34^

As to immune infiltration, M2 macrophage‐mediated tissue repair and CD8+ T cells play important roles in AMI. A previous study confirmed that in AMI, the expression of IL1R2 in M2‐type macrophages was elevated.^35^ Kolbus et al. investigated two subsets of activated CD8+ T cells as predictors of AMI and ischemic stroke during a 15‐year follow‐up.^36^

This research highlights anoikis as a target for myocardial protection during AMI. However, there were several limitations in our study. First, only two datasets were used in this study; it is difficult to add a verification set to eliminate the differences in population and genetic characteristics, and the results would be more convincing if additional relevant datasets were combined. Second, only bioinformatics was used to analyse the central genes and their potential functions related to the occurrence of AMI, and additional biological experiments are needed to verify the specific mechanisms of the screened central genes. Although there is no confirmed clinical evidence to support DsiDB as a predictive agent, interfering with anoikis may extend the lives of AMI patients. However, subsequent research is needed to analyse single‐cell sequencing data from a larger population to better comprehend this phenomenon. However, subsequent studies are needed to analyse single‐cell sequencing data from a larger population to better comprehend this phenomenon.

## 5 CONCLUSION

Through WGCNA, the key modules and key genes related to AMI were identified; the importance of these modules in AMI was further revealed; 10 hub genes (IL1B, ZAP70, LCK, FASLG, CD4, LRP1, CDH2, MERTK, APOE and VTN) were screened out. IL1B was correlated with immune cell infiltrates (macrophages, mast cells, neutrophils and T cells) in MI and most common predicted medications were roxithromycin, NSC267099 and alsterpaullone.This study provides a research basis for exploring the potential regulatory targets and possible regulatory mechanisms of AMI and offers new ideas for the treatment of this disease.

## AUTHOR CONTRIBUTIONS

**Puwei Song:** Conceptualization (equal); formal analysis (equal). **Yasen Yakufujiang:** Conceptualization (equal); formal analysis (equal). **Jianghui Zhou:** Formal analysis (equal). **Gu Shaorui:** Writing – original draft (equal); writing – review and editing (equal). **Wenli Wang:** Writing – original draft (equal); writing – review and editing (equal). **Zhengyuan Huo:** Writing – original draft (equal); writing – review and editing (equal).

## FUNDING INFORMATION

This study was funded by Excellent Discipline Reserve Talent Program of Tongji Hospital, Tongji University(HBRC1907), Specialized Program for Clinical Research in Health Industry of Shanghai Municipal Health Commission(20184Y0074) and Specialized Program for Clinical Research in Health Industry of Shanghai Municipal Health Commission(20224Y0345).

## CONFLICT OF INTEREST STATEMENT

No conflicts of interest.

## Data Availability

The data that support the findings of this study are available from the corresponding author upon reasonable request.
